# A property-based analysis of human transcription factors

**DOI:** 10.1186/s13104-015-1039-6

**Published:** 2015-03-14

**Authors:** Shahram Bahrami, Rezvan Ehsani, Finn Drabløs

**Affiliations:** Department of Cancer Research and Molecular Medicine, Norwegian University of Science and Technology, P.O. Box 8905, , NO-7491 Trondheim, Norway; St. Olavs Hospital, NO-7006 Trondheim, Norway

**Keywords:** Transcription factor, DNA-binding domain, Protein–protein interaction, Post-translational modification, Enrichment analysis

## Abstract

**Background:**

Transcription factors are essential proteins for regulating gene expression. This regulation depends upon specific features of the transcription factors, including how they interact with DNA, how they interact with each other, and how they are post-translationally modified. Reliable information about key properties associated with transcription factors will therefore be useful for data analysis, in particular of data from high-throughput experiments.

**Results:**

We have used an existing list of 1978 human proteins described as transcription factors to make a well-annotated data set, which includes information on Pfam domains, DNA-binding domains, post-translational modifications and protein–protein interactions. We have then used this data set for enrichment analysis. We have investigated correlations within this set of features, and between the features and more general protein properties. We have also used the data set to analyze previously published gene lists associated with cell differentiation, cancer, and tissue distribution.

**Conclusions:**

The study shows that well-annotated feature list for transcription factors is a useful resource for extensive data analysis; both of transcription factor properties in general and of properties associated with specific processes. However, the study also shows that such analyses are easily biased by incomplete coverage in experimental data, and by how gene sets are defined.

**Electronic supplementary material:**

The online version of this article (doi:10.1186/s13104-015-1039-6) contains supplementary material, which is available to authorized users.

## Background

Transcription Factors (TFs) are proteins that in most cases bind to specific DNA sequences known as Transcription Factor Binding Sites (TFBSs), in particular in enhancer regions or in promoter regions near their target genes [1]. The transcription factors modulate transcription initiation and regulate gene expression, and are thereby an essential part of the general regulatory system of any cell. Normally regulation of gene expression involves the binding of multiple transcription factors to the regulatory regions of a given gene. However, the definition of TFs is not always very clear-cut, and may include DNA-binding proteins that do not recognize any specific DNA motif, proteins that do not bind DNA, but influence transcription through protein–protein interactions (PPIs), and proteins that influence transcription in more indirect ways, for example by mediating chromatin remodeling [2].

Transcription factors are typically modular in structure, and will often contain effector domains and other domain types, in addition to (in most cases) one or more DNA-binding domains (DBDs). A DBD is typically a protein domain with a characteristic fold that can recognize a specific DNA sequence (motif), and thereby regulate transcription of specific target genes, although there are also examples of TFs with a more general (less motif-specific) affinity to DNA [3,4]. The interaction between a TF and its TFBSs defines the specificity of the TF, which is mediated by non-covalent interactions between the structural motif of the TF DBD and the surface of the DNA bases and backbone atoms [5,6].

Most TFs belong to one of two major classes; the general TFs and the site-specific TFs. The general TFs are important components of the basal transcriptional machinery around transcription start sites. The general TFs cannot stably bind to promoter or enhancer regions on their own. In most cases they are bound to regulatory regions through interaction with site-specific DNA-binding TFs. These site-specific TFs bind to DNA through their DBDs, and at the same time they bind to other transcriptional regulatory proteins via effector domains [7], thereby stabilizing the whole complex.

Protein–protein interactions are important for the function of proteins and the processes they are involved in, and such interactions are often facilitated by specific protein domains interacting with each other. Therefore, understanding protein interactions at the domain level can provide a generalized understanding of protein interaction, and thereby protein function. As an example, Gao *et al.* constructed a protein–protein network of transcription factors involved in regulation of liver cell proliferation and regeneration [8]. They identified 64 interactions in a regulatory network, providing additional information on the regulatory aspects of liver regeneration.

An important group of regulatory mechanisms available to the cell is post-translational modifications (PTMs). The PTMs are highly dynamic and often reversible, and they may occur on almost all proteins. Most PTMs change the properties of a protein by the addition of a specific chemical group to one or more of its amino acid residues [9,10]. The PTMs make possible diverse signaling that is suitable for relaying rapid messages throughout the cell. Some PTMs, such as phosphorylation, can be quite transient, and may serve to rapidly activate or deactivate a protein, whereas other PTMs may be more long-lasting. PTMs may create further signaling through modular protein domains that recognize particular types of PTMs located on specific residues. A relevant example of how PTMs may modify TF function is the MEF-2A factor which regulates gene expression in neuronal cells, where it can act as either a transcriptional activator or a repressor. This switch is controlled by post-translational modification of MEF-2A, with acetylated MEF-2A acting as a transcriptional activator, whereas the factor acts as a transcriptional repressor when it is modified by sumoylation and phosphorylation [11].

This shows that the regulatory roles of TFs can be modified by the properties of the TFs, including DNA-binding and effector domains, PPIs and PTMs. Therefore there is a need to increase our knowledge about TF domains and other properties, in addition to their binding sites in target genes, and this makes a collection of well-curated annotation data of TFs highly relevant.

There are some existing TF databases, but in general they contain very limited information about TF properties, except for DNA motif specificity, most often through a Position Weight Matrix (PWM), and links to more general protein databases with additional information. For example, JASPAR is an open-access database of DNA binding site profiles, based on collections of position frequency matrices (PFMs) that are mainly derived from published data, including chromatin immunoprecipitation and sequencing (ChIP-seq) experiments. The newest JASPAR version includes interfaces to several packages (BioPython, Rtool, R/Bioconductor) to facilitate access for both manual and automated methods [12,13].

Zhang *et al*. published in 2012 a comprehensive animal transcription factor database based on DNA-binding domains, where they collected and curated 71 animal TF families [14]. Although this includes detailed annotations for each TF (basic information, gene structure, functional domain, 3D structure hit, Gene Ontology, pathway, protein–protein interaction, paralogs, orthologs, potential TF-binding sites and targets), it is not very suitable for detailed analysis of TF properties. Fulton *et al*. made in 2009 a catalog of mouse and human TFs (called TFCat), where TFs were classified according to evidence supporting DNA-binding and transcriptional activation [15]. TFCat was based on information from four transcription factor data sets, and categorized DNA-binding TFs into 9 protein groups with 39 protein families. It is a very useful resource for TF classification, but with limited information on TF properties. Vaquerizas *et al.* used a set of 1391 manually curated sequence-specific DNA-binding transcription factors to investigate function, genomic organization and evolutionary conservation [16]. Ravasi *et al*. identified almost 2000 proteins from the human genome that are potential TFs [17]. They built a global atlas of combinatorial transcriptional regulation in mouse and human and screened for physical interactions between the majority of human and mouse DNA-binding transcription factors. This is again a useful resource, but with limited additional information.

In this paper we describe the collection and curation of a list of properties for human TFs, using the list of TFs published by Ravasi *et al*. The main reason for using this particular data set was that it also includes a consistent set of protein–protein interaction data, with a clear distinction between missing data and lack of interaction. The properties that were added include DNA-binding domains, protein–protein interactions, and post-translational modifications. We then show how this can be used for example to identify sub-groups of TFs and to correlate these with specific functions, and to identify TF properties that are associated with specific processes. However, we also show that such analyses are easily biased by data set composition and incomplete annotations, and therefore have to be interpreted with great care. The TF property data set and software for data analysis is available with the paper as additional data.

## Methods

### Initial definition of a data set of human TFs

We used a list of 1988 human transcription factors, originally used by Ravasi *et al*. to build an atlas of combinatorial transcriptional regulation [17]. The gene names were checked against HGNC [18] and UniProt [19], and duplicates were removed. This gave a final list of 1978 TFs. Initial annotation of the TFs was based on database entries downloaded from UniProt (last update done using release 2012_07).

### Comparison to other TF collections

The gene list from Ravasi *et al.* was compared to previously published gene lists from Zhang *et al.* [14] and Vaquerizas *et al.* [16]. These additional gene lists were downloaded from supplementary material. DAVID does not accept HGNC gene names for explicit definition of background, therefore the gene names were remapped to UniProt IDs for DAVID analysis, using the ID converter of BioMart (http://www.biomart.org/) [20].

### General domain annotation

Specific domains, as defined for example in Pfam [21], are often associated with specific functions, and are therefore an important annotation resource. Unfortunately the Pfam annotation in UniProt does not include information about sequence position of Pfam domains. Therefore we downloaded the most recent swisspfam list from Pfam (last update done using release 12.03.2013), and searched the list for UniProt IDs [19,21].

Our annotation data include both levels of Pfam families; Pfam-A and Pfam-B. Both entry types are made from the most recent release of UniProtKB at a given time and produced automatically from the non-redundant clusters after sequence clustering. Pfam-A entries can be successfully annotated by profile HMM searches of primary sequence databases, whereas Pfam-B entries are un-annotated [21].

### Adding annotation on DNA-binding domains

In the following description we try to distinguish between the domains as defined by Pfam (*Pfam domains*), and the individual occurrences of these domains in a set of proteins (*domain occurrences*). In order to add annotation on Pfam domains acting as DNA-binding domains (DBDs), all entries for Pfam domains assigned to the list of TFs were first manually reviewed and curated for evidence strongly suggesting DNA binding, using Pfam descriptions and associated literature references. In order to get a more complete annotation of DBDs in these proteins, we then used a DBD prediction method to identify additional Pfam domains as DNA-binding. In order to distinguish between sporadic and consistent predictions we did the DBD predictions over all Pfam domains in the set of TF proteins, including domains assumed not to be DNA-binding. We then estimated the overall prediction quality over all occurrences for each Pfam domain, on the hypothesis that it was a DBD, and used a support vector machine (SVM) [22] to distinguish between true positive and false positive cases. Ideally, Pfam domains where individual occurrences frequently overlap with DBD predictions should be accepted as true positive cases, whereas Pfam domains with few overlaps should be rejected as false positives. The challenge is to find a suitable cutoff between these two alternatives.

We used the threading-based method DBD-Threader [23] for the prediction of DNA-binding domains. In this method DNA-binding propensity is calculated using a statistical DNA–protein pair potential. The sequence of a target protein is compared against an experimentally determined template library of DNA-binding protein domains, using threading. Any significant template hits are further evaluated using the DNA–protein interaction energy, calculated using the alignment of the target template and the corresponding DNA structure in complex with the template protein. If there is at least one significant template for a target protein according to the specified Z-score and energy threshold conditions, the protein is predicted to be DNA-binding, otherwise it is classified as non-DNA-binding [23]. It has been shown that DBD-Threader has significantly improved performance when both threading Z-score and protein–DNA interaction propensity are taken into account, leading to a sensitivity of 56% and a precision of 86% on a benchmark set with 179 DNA-binding and 3797 non-DNA-binding proteins [23]. The method has also shown good performance in an independent benchmark study, in particular with respect to specificity [24].

We used a reference set of TFs with Pfam domains where we knew from manual curation that these specific Pfam domains were DNA-binding. On this set we predicted DBDs using DBD-Threader. We then compared annotated and predicted DNA-binding regions, and estimated the quality of the predictions at three different levels; protein level, domain level, and residue level, in order to find optimal criteria for identifying false positive predictions.

#### The protein level

At this level we predicted whether a protein was DNA-binding or not, irrespective of domain overlap. We used the set of proteins where curated annotation data showed that they were DNA-binding because they contained a Pfam domain annotated as DNA binding [Additional file 1]. We then counted the number of TFs with a known DBD that also were predicted to have a DBD, and estimated the rate of true positive predictions, or sensitivity (Sn, Equation 1).

1 $$ \mathrm{S}\mathrm{n} = \mathrm{T}\mathrm{P}/\ \left(\mathrm{T}\mathrm{P} + \mathrm{F}\mathrm{N}\right) $$

#### The domain level

**Figure 1:**
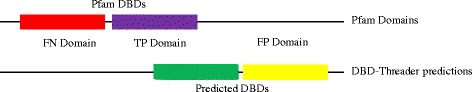
**Prediction quality at the domain level.** Domains are classified as TP, FN and FP as shown, relative to the curated Pfam domains. TNs are not included in this comparison, as negative domains are not well defined.

At the domain level we tested how often the predicted DBD (for proteins correctly predicted to have a DBD) showed overlap with the known DBD (from curated annotation data), see Figure 1 for details. For each known DBD we compared it to the predicted DBD and estimated the amount of overlap relative to the Pfam domain. An overlap of at least 1 residue was counted as significant, and the values for TP, FN and FP were used to estimate sensitivity (Sn, Equation 1) and positive predictive value (PPV, Equation 2).

2 $$ \mathrm{P}\mathrm{P}\mathrm{V} = \mathrm{T}\mathrm{P}/\ \left(\mathrm{T}\mathrm{P} + \mathrm{F}\mathrm{P}\right) $$

#### The residue level

**Figure 2:**
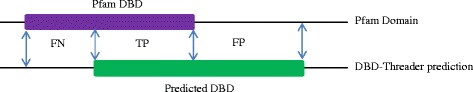
**Prediction quality at the nucleotide level.** Regions are classified as TP, FN and FP as shown, relative to overlap with the curated Pfam domains. TNs are not included in this comparison, as they represent a very large fraction of the comparison, which may bias the analysis.

At the residue level we measured the amount of overlap between known and predicted DBDs for the actual overlaps that were identified above. This was done according to Figure 2, and used to estimate Sn and PPV as for the domain level.

#### Predicting new DBDs

DBD-Threader was run on all TFs, and occurrences of Pfam domains showing any overlap with DBD predictions were used as an indication of potential DNA-binding. In order to distinguish between random overlaps and true DBDs we used the Support Vector Machine method (SVM) [22] as implemented in scikit-learn version 0.15.0 [25], with a linear kernel function, and used it to separate false positive from true positive cases, based on prediction quality according to the hypothesis that each Pfam domain is a DBD. The Pfam domains annotated as DBDs after manual curation were considered as positive data, and for negative data we identified any additional Pfam domains in the DNA-binding proteins with at least one known DBD, arguing that most likely the majority of the remaining domains of these proteins are non-DBDs. These Pfam domains were evaluated by manual curation (scientific literature and Pfam entry annotation), and were separated into 2 groups; Pfam domains with *unknown* DBD status, and *non-DBD* Pfam domains [Additional file 1]. Obviously, only non-DBD Pfam domains that showed some overlap with DBD-Threader predictions could actually be used as negative data for the SVM classifier. Initial tests showed that the SVM had best performance on data at the residue level, leading to better separation of positive and negative cases (data not shown), so we used residue level %Sn and %PPV as features for classification. We then determined the final set of DBDs based on the SVM output.

### PTM annotation

For data on post-translational modifications (PTMs) we used information from PhosphoSite (last update done using release 01.01.2014) [26]. We imported data for 6 PTM types; acetylation, methylation, O-GlcNAc, phosphorylation, sumoylation and ubiquitination.

### GOrilla and DAVID

We used GOrilla [27,28] and DAVID [29] for enrichment analysis of TF subsets on a broad range of annotation data. The reason for using both tools is that although DAVID can analyze a broader range of properties, the information in GOrilla is more up to date. In general we used a specific subset as the positive set, and the full set of TFs as background. In cases where we could identify the subset of TFs for which we had reliable data (e.g. the PPI data) we used this subset as background. In most cases (e.g. for PTMs) it was difficult to identify TFs for which we actually had a lack of data (rather than negative data), and in these cases the full TF set was used.

### Protein–Protein Interactions

Ravasi *et al*. were able to capture cDNA clones for 1222 TFs in human, in order to map PPIs [17]. The number of possible interactions (including homo-dimers) is $\frac{n\left(n+1\right)}{2}=\frac{1222\kern0.5em \times 1223}{2}=747253,$ but based on the data from Ravasi *et al*. only 762 out of these (0.1%) were observed as actual interactions. This set was tested for correlation against other features, using a general enrichment analysis.

### Enrichment analysis

The enrichment analysis was implemented as a Fisher’s exact test on a 2 × 2 contingency table. Observations were grouped according to pairs of properties, like being involved in PPIs (yes/no) and having a DBD (yes/no). This was then tested using the Fisher’s exact test, in most cases with a threshold for p-value at 0.05 after Benjamini correction for multiple testing. In addition to the p-value, the expected number of occurrences and the Matthew’s correlation coefficient (MCC, Equation 3) was estimated for cases with significant p-values. The testing was implemented using the full set of TFs (1978) as background for all properties except PPI. For the PPI case we used the 1222 TFs actually mapped for PPI in the Ravasi *et al*. paper as background. For calculation of MCC, a TF was considered as TP if it had both properties, as TN if it had none of properties and as FN or FP if just had one of the properties (based on the 2 × 2 contingency table).

3 $$ \mathrm{M}\mathrm{C}\mathrm{C} = \left(\mathrm{T}\mathrm{P}\times \mathrm{T}\mathrm{N}\ \hbox{--}\ \mathrm{F}\mathrm{P}\times \mathrm{F}\mathrm{N}\right)/\ \mathrm{sqrt}\ \left(\left(\mathrm{T}\mathrm{P} + \mathrm{F}\mathrm{P}\right)\ \left(\mathrm{T}\mathrm{P} + \mathrm{F}\mathrm{N}\right)\ \left(\mathrm{T}\mathrm{N} + \mathrm{F}\mathrm{P}\right)\ \left(\mathrm{T}\mathrm{N} + \mathrm{F}\mathrm{N}\right)\right) $$

Python scripts were used to extract subgroups of TFs with specific properties for enrichment analysis [30]. Biopython was used to extract all gene names for each TF from the UniProt files [31]. The p-values were estimated using the Fisher 0.1.4 package [32]. The software for enrichment analysis is available with the paper.

### Ethical approval and consent

This study is based on human data. However, all data have been downloaded from open data repositories (UniProt, Pfam, PhosphoSite) or from supplementary material from existing publications (see text), and cannot be linked to individuals. Ethical approval and consent is therefore not required.

## Results and discussion

### Making an initial set of TFs

The starting point for the annotated TF list was the set of 1988 TFs by Ravasi *et al*. [17]. These TFs were then supplemented with annotation data as described below and in Methods, in particular with respect to UniProt IDs, Pfam domains including DBDs, PPI data and PTMs.

### Comparison to other TF collections

We wanted to use the data set by Ravasi *et al.* in order to utilize the consistent set of PPI data generated for that particular data set. However, alternative data sets have been used in other studies, and in order to put the set from Ravasi *et al.* into context, we compared it to the sets from Zhang *et al.* [14] and Vaquerizas *et al.* [16]. The set by Zhang *et al.* is based on manual curation of animal TF families, and includes a separation into DNA-binding TFs, TF cofactors and chromatin remodeling factors. The set by Vaquerizas *et al.* is based on curation of a list of potential TFs identified from InterPro database entries.

We first tested for overlap between the different lists based on unique HGNC gene names (see below). This showed a quite similar overlap of 1253 genes between Ravasi and Vaquerizas, 1374 between Ravasi and Zhang, and 1404 between Vaquerizas and Zhang. These numbers are on average 10% lower if we focus on DNA-binding TFs (1132, 1100, and 1359, respectively (see below for definition of DBDs in the Ravasi set)). Of the genes included in the Ravasi set, 186 and 66 are classified in the Zhang set as TF cofactors and chromatin remodeling factors, respectively. This overlap is reduced to just 14 and 10 if we focus on DNA-binding TFs in the Ravasi set.

The similarity between the data sets from Ravasi and Vaquerizas is further confirmed by comparing the distribution of domain types. The Vaquerizas set is strongly dominated by the InterPro domains ZNF-C2H2, Homeodomain, HLH and bZip, in that order. This is very similar to the distribution of Pfam domains in the Ravasi set (see below for how they were mapped), which is dominated by the Pfam domains for zinc fingers, homeobox, HLH and bZIP (Figure S1 [see Additional file 2]). The Ravasi set may be somewhat enriched in rare Pfam domains (i.e. domains found less than 5 times), but this may also be caused by differences between InterPro and Pfam.

In order to highlight the differences between these collections we used unique genes from each collection as input to DAVID and GOrilla, in each case using the full gene list for that collection as background. The genes that are unique to Ravasi compared to Vaquerizas are enriched for histone-related properties and transcription co-factor activity (results not shown), indicating that it contains some cases that are not classical TFs. The Vaquerizas set is, on the other hand, enriched for RNA binding activity, but also catalytic activity, indicating that also this data set may contain cases that are not TFs according to a strict definition. Comparison of the Ravasi data to the Zhang data shows a similar pattern, with some enrichment for RNA binding and histone-related properties in the Ravasi set. This shows that the gene set defined by Ravasi *et al.* may have some inherent biases, but that this may be a problem also in other gene sets.

### Mapping of UniProt IDs and Pfam domains

The gene names by Ravasi *et al.* were mapped to unique HGNC and UniProt IDs. In total 1978 TFs (99.5%) could be mapped to unique IDs. Mapping of Pfam domains was done using the annotations from Pfam (in swisspfam) [21]. The list of 1978 human TFs had 1664 unique Pfam domains, which included 936 Pfam-B domains and 728 Pfam-A domains. However, most of the Pfam domains have few occurrences in the set of human TFs (see later).

### Mapping of DBDs

#### Verification on known Pfam DBDs

The ability for motif-specific DNA binding is an important property of most TFs. However, it is not necessarily an essential property, as TFs also can interact through PPIs. The observation of TFs that may bind to regions without any apparent binding site motifs highlights this. Motif-specific vs motif-less binding may have functional relevance, and it is therefore important to identify TFs with and without DNA-binding domains.

Less than 1% of all proteins have an experimentally determined structure, which makes it difficult to assign function based on structure. However, significantly similar sequences may share function, although functional roles of related proteins can change during evolution [33]. Therefore prediction methods based on sequence/structure similarity can be used to try to identify DNA-binding domain types when annotation is lacking. However, such predictions will contain some false positive and false negative predictions. It is difficult to correct for false negative predictions, i.e. to recognize something that was missed by the prediction method. However, it may be possible to correct for false positive predictions by estimating prediction quality over a set of predictions. Here we used Pfam domains as a basis, and tried to predict individual occurrences of DNA-binding for these Pfam domains. We could then estimate the consistency of prediction over all occurrences of a given Pfam domain as a quality measure, and use this to identify predictions that are likely to be false positive.

As a first step the 728 Pfam-A entries were checked for DNA-binding properties from scientific literature and Pfam entry annotation. This showed that after manual curation 70 of the Pfam-A domains were confirmed to be DNA-binding [see Additional file 1], and the proteins that had at least one of these DNA-binding domains were classified as DNA-binding proteins. These 70 DNA-binding Pfam domains were found in 907 proteins, whereas 1071 proteins did not have a reliably annotated DNA-binding domain at this stage.

We then used DBD-Threader to predict additional Pfam domains as DBDs [23] (please see Methods for details). As an initial estimate of the expected reliability of predictions, we started by doing prediction on the 907 TFs with known DBDs. These predictions were evaluated at three different levels. At the *protein level* we just checked whether the protein was predicted to be DNA binding or not. This may be useful for classification of TFs, but it does not identify new DNA-binding domains. Therefore, for the true positive predictions at the protein level we also evaluated the predictions at the *domain level*, by checking whether the prediction was able to identify the correct Pfam domain as DBD. This was evaluated both for each domain type, and over all domain occurrences. For the true positive predictions at the domain level, we finally evaluated the predictions at the *residue level*, by checking how well the predictions overlap with the Pfam domain annotated as DBD. The results (Table 1) showed that 776 out of the 907 TFs had been correctly predicted by DBD-Threader as DNA-binding. At the domain level, 40 out of the 70 known DNA-binding domains were correctly predicted by DBD-Threader at least 50% of the time, giving a sensitivity of 57%. We then considered the domains with correct prediction frequency of less than 50% as FN domains. Statistics based on domain occurrences rather than domain types gave a higher sensitivity (74%), showing that performance is better on frequently occurring domains. Doing the statistics at the level of residues gave a somewhat lower sensitivity (62%). The most likely reason for this is shown in the average values, with a relatively high FN rate. This shows that the Pfam domains on average are longer than the predicted DBDs.

**Table 1:** **Prediction results for DNA-binding domains on positive data**

**Level**	**Unit**	**NPfam**	**Npredicted**	**TP**	**FP**	**TN**	**FN**	**Sn**	**PPV**
Protein	proteins	907	776	718	-	-	189	79.16	-
Domain	domains	70	46	40	-	-	30	57.14	-
Domain	occurrences	1159	872	863	519	-	296	74.46	62.45
Nucleotide total	nucleotides	69320	43326	42783	16899	-	26537	61.72	71.68
Nucleotide average	nucleotides	59	49	49	32	-	89	35.51	60.49

The results in Table 1 show that DBD-Threader in general works quite well, with sensitivity of almost 75% for the identification of DNA-binding domains. In particular it seems to work well for frequent DBDs, which means that a large fraction of DBD-containing proteins will be correctly identified, whereas rare cases are more likely to be missed.

**Figure 3:**
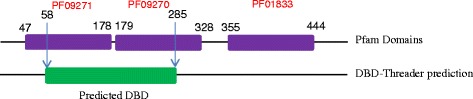
**Example of a challenging DBD prediction.** The predicted region overlaps with two independent Pfam domains.

Some predictions were checked in more detail, based on high FP/FN rates or large differences in Sn and PPV. This involved three domain types (LAG1-DNAbind (PF09271), BTD (PF09272), and HNF-1_N (PF04814)), and two of these (PF09271 and PF09272) did illustrate a potential problem, as there was one predicted continuous DBD overlapping two Pfam domains (Figure 3). This gives a low overlap when each domain is treated individually. The manual evaluation also showed that the HNF-1_N domain is likely to be an outlier. However, this constitutes a small fraction of the actual domains, and has minor impact on the analysis.

#### Identification of additional DBDs

For identifying additional Pfam domains as DBDs we used DBD-Threader predictions as a starting point. We then used the average overlap over all occurrences of each Pfam domain as input for a Support Vector Machine (SVM) [22], in order to identify Pfam domains that had too low overlap with DBD predictions to be classified as DNA-binding. As positive data we used the 40 Pfam domains that were correctly predicted by DBD-Threader as DNA-binding. As negative data we used any additional Pfam domains co-occurring with the 40 Pfam domains in the positive set [Additional file 1], based on the assumption that most TFs only have one type of DBD. This may be an oversimplification in some cases, but the SVM approach is supposed to be robust with respect to outliers. The negative data also had to show some overlap with DBD-Threader predictions in order to be useful for defining a classification cutoff between true positive and false positive cases (all Pfam domains without any overlap with DBD predictions will be zero in both Sn and PPV). This left only 6 Pfam domains as negative data. However, this should be a reliable data set of non-DBD Pfam domains in DNA-binding proteins, despite the small size.

The SVM classifier was used with the %Sn and %PPV values for DBD-Threader predictions on each Pfam domain, over all occurrences (i.e. for the hypothesis that the Pfam domain is a DBD). The performance of the classifier was assessed on the 46 Pfam domains with known classification by using a two-way cross-validation with five re-samplings, in addition to a leave-one-out cross-validation. This gave an average performance of 98% for both Sn and PPV. We then used this SVM to classify the remaining Pfam domains, based on overlap (or lack of overlap) of individual occurrences of each domain with the DBD-Threader predictions (Figure S2 [see Additional file 2]). For prediction of new DBDs we focused on Pfam-A domains, and 38 Pfam domains not included in the training set showed a non-zero overlap with DBD-Threader predictions. According to the SVM step 27 of these Pfam domains could be reliably identified as DNA-binding whereas 11 Pfam domains were more likely to be non-DNA-binding (Table 2).

**Table 2:** **New DNA-binding and non-DNA-binding domain types**

**DBD**	**DBD**	**DBD**	non-DBD*
Homeobox_KN	zf-C2H2_6	Maf1	PBC
MCM2_N	zf-C2H2_4	zf-H2C2_5	zf-C2H2_2
CBFD_NFYB_HMF	TFIID-18 kDa	Exo_endo_phos	TFIIA
SKIP_SNW	TFIIB	DUF3432	SCAN
Ku	DNA_methylase	Toprim	Prox1
Pax2_C	TFIID_20kDa		SSXRD
TAFII28	ResIII		HJURP_C
DUF2028	FAD_binding_7		Ku_N
Histone	RNA_pol_Rpb1_1		DNA_photolyase
zf-H2C2_2	SOXp		SNF2_N
zf-met	DNA_topoisoIV		TIG

*After filtering predicted DBDs for false positives.

Following the above analysis we had in total 97 Pfam-A domains annotated as DNA-binding, including the 30 domains that were annotated as DBD in literature, but not reliably predicted by DBD-Threader in the initial analysis. A total of 1225 proteins had at least one occurrence of a Pfam domain annotated and/or classified as DBD, and were therefore considered to be DNA-binding, whereas the remaining 753 proteins could not be identified as DNA-binding. This means that at least 61% of the TFs are DNA-binding, and this number seems to be comparable to the result from Fulton *et al.* [15].

Pfam-B domains were not included in the final prediction process for new DBDs. Such domains are generated by an automatic process, which means that they do not have a stable definition, and they will often be of low quality. Also, they had only minor impact on the actual TF classification. 45 Pfam-B domains showed at least some overlap with DBD-Threader predictions. Following the SVM-based analysis 25 out of them were confirmed as DNA-binding, whereas 20 Pfam-B domains were identified as non-DNA-binding. The 25 possibly DNA-binding Pfam-B domains were found in 27 TFs, but 24 of these TFs had at least one DNA-binding Pfam-A domain, and had therefore already been identified as DNA-binding TFs.

The number of TFs with a clear DBD is certainly a conservative estimate, as DBD-Threader could not reliably identify all Pfam domains that are known DBDs according to literature annotation. However, as we also have shown that this affects mainly the less frequently occurring DNA-binding domains, we believe that the estimate is at least close to the real value.

### Mapping of PPIs and PTMs

**Figure 4:**
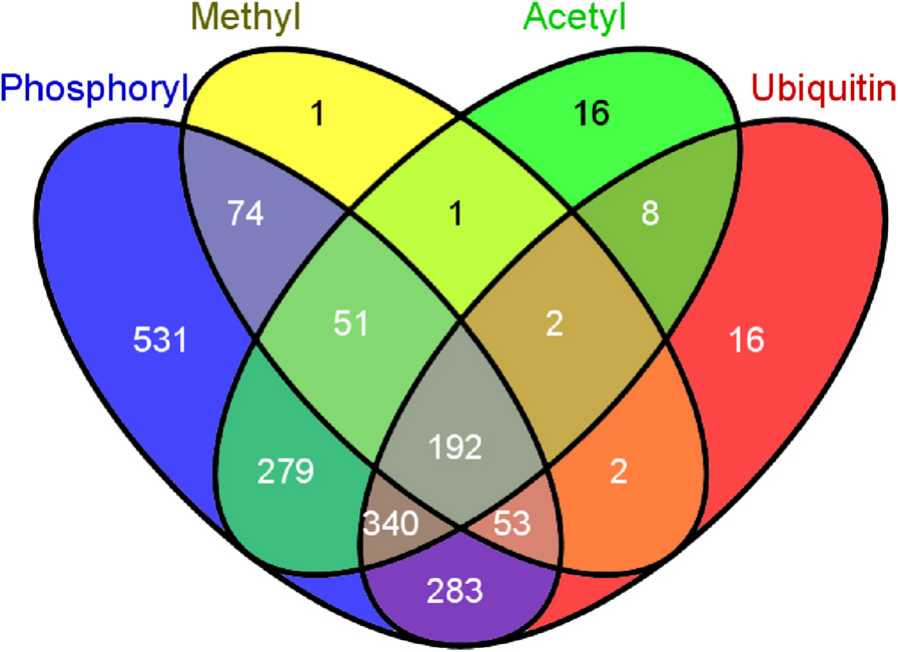
**A Venn diagram for distribution of PTMs across TFs.** The diagram shows that PTMs tend to co-occur, possibly due to experimental bias.

Ravasi *et al.* tested 1222 TFs experimentally for protein–protein interactions and found 762 actual interactions for 482 TFs [17]. These interactions were included in the data set. For the mapping of PTMs, we retrieved information for each TF from the PTM-specific files from Phosphosite [26]. The distribution of PTMs is shown in Figure 4.

Based on these data sources, including the analysis of DBDs described above, we then made a final annotated set of transcription factors. The main properties are listed in Table 3, and the full table is available [see Additional file 3].

**Table 3:** **Overview of TF annotation data**

**Information**	**Type**	**TFs with data**	Positives*	Average**
Uniprot ID	protein ID	1978	1978	1
Pfam non-DBD	domain IDs	1978	753	2.16
Pfam DBD	domain IDs	1978	1225	1.33
PPI	protein IDs	1222	482	1.58
PTM - acetylation	positions	1978	884	3.55
PTM - methylation	positions	1978	376	3.22
PTM - O-GlcNAc	positions	1978	41	2.90
PTM - phosphorylation	positions	1978	1797	13.12
PTM - sumoylation	positions	1978	190	1.77
PTM - ubiquitination	positions	1978	896	4.38

*Number of TFs that actually have the property. **Average number of occurrences in the positive TFs.

### Using the annotated TFs for data analysis

We now want to illustrate how such data can be used to analyze sets of TFs. We used two main approaches. In the first approach we used properties in the TF table to split the set of TFs into subsets, and analyzed these subsets using either enrichment analysis against other properties in the TF table, or against Gene Ontology data or annotation-based property data, using GOrilla [27,28] and DAVID [29]. As a more general approach we also used external data to define subsets of TFs, and then analyzed these subsets using enrichment analysis against properties in the TF table.

#### Subsets analyzed with GOrilla and DAVID

Here subsets were defined based on properties in the TF table, like DNA-binding or acetylation, and these subsets were analyzed with GOrilla and DAVID, using the full set of relevant TFs as background. Selected results for GOrilla are shown in Table 4, and comprehensive results for GOrilla and DAVID are given in Table S1 and S2 [see Additional file 2].

**Table 4:** **Selected enriched terms according to GOrilla**

**Description**		**P-value**	**FDR q-value**	**Enrichment (N, B, n, b)**
DNA_Binding	DNA binding	2.11E-185	1.72E-182	1.28 (1939,1475,1206,1174)
	core promoter sequence-specific DNA binding	7.87E-5	1.79E-3	1.37 (1939,60,1206,51)
	protein dimerization activity	4.00E-8	1.13E-6	1.24 (1939,254,1206,196)
Non_DNA_Binding	catalytic activity	1.07E-49	8.75E-47	2.01 (1939,305,735,232)
	RNA binding	3.95E-34	1.62E-31	2.00 (1939,222,735,168)
	transcription cofactor activity	9.56E-12	4.61E-10	1.42 (1939,359,735,193)
	histone binding	1.03E-10	3.39E-9	2.07 (1939,60,735,47)
	ubiquitin-protein transferase activity	2.29E-10	7.21E-9	2.40 (1939,33,735,30)
	methylated histone binding	3.80E-10	1.11E-8	2.54 (1939,26,735,25)
Acetylation	transcription factor binding	2.12E-6	2.17E-4	1.28 (1939,292,879,169)
	structure-specific DNA binding	2.27E-5	7.76E-4	1.38 (1939,136,879,85)
Non_Acetylation	sequence-specific DNA binding	1.36E-6	1.11E-3	1.11 (1939,887,1061,537)
Methylation	protein binding	2.67E-8	3.12E-6	1.21 (1939,1135,372,264)
	chromatin binding	3.93E-7	2.48E-5	1.62 (1939,264,372,82)
O-GlcNAc	protein binding	6.83E-6	2.80E-3	1.54 (1939,1133,41,37)
	histone deacetylase binding	2.71E-4	7.41E-2	6.31 (1939,45,41,6)
Phosphorylation	protein binding	4.93E-5	2.02E-2	1.02 (1939,1133,1782,1065)
PTM	protein binding	3.12E-6	2.55E-3	1.02 (1939,1135,1827,1093)
Sumoylation	sequence-specific DNA binding	3.00E-12	4.1E-10	1.73 (1939,617,189,104)
	core promoter binding	1.86E-7	8.03E-6	2.90 (1939,92,189,26)
	chromatin binding	1.92E-7	7.86E-6	1.98 (1939,264,189,51)
Ubiquitination	protein binding	3.71E-30	3.04E-27	1.24 (1939,1133,888,641)
	transcription cofactor activity	3.27E-8	8.12E-7	1.28 (1939,359,888,211)
Non_Ubiquitination	DNA binding	6.99E-14	5.73E-11	1.09 (1939,1473,1052,869)
PPI	transcription factor binding	1.38E-4	4.83E-2	1.31 (1203,185,475,96)

The results show a particularly clear difference between TFs with and without a DBD. The DNA-binding TFs are enriched in sequence-specific DNA-binding, receptor properties, dimerization and core promoter interactions. The non-DNA-binding TFs are enriched in RNA-binding and cofactor activity, but also in catalytic activity, histone binding and related processes. This shows that the list of TFs includes some epigenetic factors. In order to verify this we compared the TF list used here to a list of epigenetic factors (F. Drabløs, unpublished data). This indicates that the list included 322 genes (16%) that also could be classified as epigenetic factors. This is probably an overestimate, as the list of epigenetic factors includes some TFs that recruit epigenetic factors. However, it confirms that subsets of genes on the list from Ravasi *et al.* are not classical TFs.

#### Associations between individual PTM properties

The modification of transcription factors by PTMs like phosphorylation, acetylation, methylation, ubiquitination, sumoylation and O-GlcNAc may affect their activity. It is therefore relevant to see how these modifications are correlated, and whether they are correlated with other properties. This is shown in Table 5, and in Table S5 [see Additional file 2].

**Table 5:** **Associations between property-based subgroups**

**Property pair**		**P-value**	**Benjamini**	**Corr.**
Phosphorylation	Acetylation	1.84E-10	5.15E-09	0.190
Phosphorylation	Ubiquitination	1.94E-10	2.72E-09	0.190
DNA_Binding	Methylation	2.08E-10	1.94E-09	−0.156
Phosphorylation	Methylation	2.42E-10	1.70E-09	0.127
Methylation	Acetylation	2.78E-10	1.56E-09	0.202
Ubiquitination	Methylation	2.85E-10	1.33E-09	0.204
DNA_Binding	Ubiquitination	3.16E-10	1.26E-09	−0.280
Ubiquitination	Acetylation	3.39E-10	1.19E-09	0.289
Acetylation	Sumoylation	5.99E-09	1.86E-08	0.131
Ubiquitination	Sumoylation	4.03E-08	1.13E-07	0.124
DNA_Binding	Acetylation	6.30E-08	1.60E-07	−0.122
Methylation	O-GlcNAc	1.24E-05	2.90E-05	0.110
Phosphorylation	Sumoylation	1.51E-05	3.24E-05	0.086
Acetylation	O-GlcNAc	3.45E-04	6.91E-04	0.083
Ubiquitination	O-GlcNAc	3.82E-03	7.13E-03	0.067
PPI	Sumoylation	1.23E-02	2.16E-02	0.072
Methylation	Sumoylation	1.49E-02	2.46E-02	0.056
Phosphorylation	O-GlcNAc	2.85E-02	4.43E-02	0.046
DNA_Binding	PPI	3.43E-01	4.37E-01	−0.027

The results show significant associations between most of the PTMs. It is likely that this shows an experimental bias in the data set, where TFs tested for a given PTM also are more likely to have been tested for other PTMs, thereby creating artificially strong associations. Figure 4 seems to indicate this, as for example almost all proteins that are methylated are also phosphorylated. We also see that there is in general a negative correlation between PTMs and DNA-binding properties, possibly indicating that PTMs are less important for classical TFs than for TFs involved for example in chromatin organization. This may indicate that processes at the chromatin level are more actively regulated at the PTM level than TF binding itself, which seems reasonable based on current knowledge.

#### Association between DNA-binding and PPI

It is relevant to look further into possible associations between DNA-binding and PPI propensity, as stabilization through PPI is a possible mechanism for stable binding despite lack of strong DBDs in TFs. As seen from Table 5, there is not any significant non-random association between having a DNA-binding domain and participating in PPI (p-value 0.343).

However, this is a rather general analysis, and it may be relevant to look closer into more specific cases, where one, both or none of the TFs have a DBD. These results are shown in Table 6. The results show that all cases are significant after Benjamini correction, in particular for cases with no DBD in any of the partners, where we see more pairs than expected. For the other two cases, where at least one TF is DNA-binding, we see fewer pairs than expected. A reasonable initial hypothesis would have been that TFs without a DBD will tend to associate with TFs with a DBD, in order to recognize regulatory regions, but this analysis indicates the opposite. The data make sense for cases where both TFs have DBD, and therefore do not need PPI to bind, but we do not have a good explanation for the other two cases, although participation in large complexes may be a possible hypothesis.

**Table 6:** **Occurrence of DBDs in 762 PPI pairs**

**DBD found in**	**Expected**	**Observed**	**P-value**	**Benjamini**
both TFs	255	229	0.046	4.58E-02
only one TF	371	343	0.042	4.58E-02
none TFs	135	190	7.50E-07	2.25E-06

#### Enrichment of domains and domain pairs in PPI

PPIs are often achieved through interactions between specific domains. It is therefore interesting to see whether specific Pfam domains, or pairs of Pfam domains, are enriched in the PPI data.

As previously described there were 762 PPIs involving 482 transcription factors, and these TFs contained 518 different Pfam domains. Each Pfam domain was tested for association with PPI. This identified 73 enriched Pfam domains [see Additional file 4].

**Figure 5:**
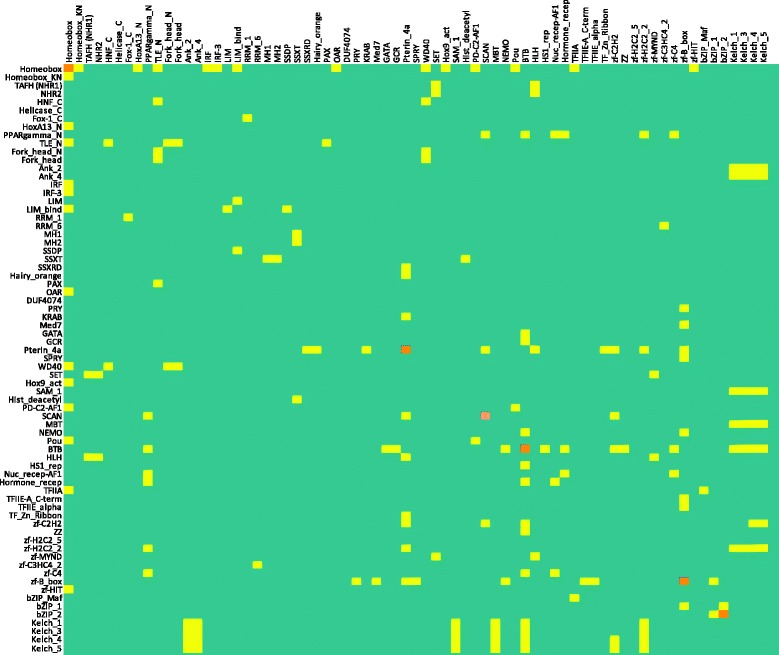
**A matrix representation of enriched domain pairs in PPI data.** Homodimers are indicated in orange. RNA polymerases and Pfam-B domains are not included; please see Figure S2 [in Additional file 2] for the full data set.

Subsequently we tested pairs of Pfam domains, rather than individual occurrences. First we tested all possible pairs for the 73 Pfam domains (see above), which identified 227 enriched pairs of Pfam domains. However, there is a risk that some interactions are significant as pairs even though they are not significant individually. We therefore relaxed the criteria so that at least one of the two Pfam domains had to be significantly associated with PPI [see Additional file 4]. In total we identified 347 pairs of Pfam domains as enriched in PPI data after Benjamini correction. However, 177 out of the 347 pairs were observed just once [see Additional file 4]. The main pairwise interactions, except for RNA polymerases and Pfam-B domains, are plotted in Figure 5. All interactions are shown in Figure S3 [see Additional file 2]. The plot shows that the network of domains that are enriched (and possibly involved) in PPI is quite sparse. Although more than half (66%) of the domain pairs are found in pair with more than one other domain type, this is in most cases limited to two different domains, and often involve related types (like Kelch domains).

### Analysis of externally defined sets of TFs

To illustrate how such annotated lists can be used to analyze data from different types of experiments, we analyzed gene lists from three recent papers. The software used for this analysis is available with the paper [Additional file 5].

A paper by Tuomela *et al.* discusses early changes in gene expression during differentiation of human Th17 cells from CD4^+^ T-cells [34]. Expression levels were measured with microarrays, and differentially expressed genes were identified. One of the largest groups of differentially expressed genes was transcription factors. Groups of genes with similar temporal changes in expression patterns were identified by clustering into 10 groups (see the paper for details). Some of these groups showed similar general trends, like groups 1, 2 and 3 (up-regulation), 4, 5 and 6 (down-regulation), and 7, 8, 9 and 10 (no change). All the individual groups, as well as the indicated combinations, were tested for enrichment [see Additional file 6]. The results (Table 7; full results in Table S4 [see Additional file 2]) show that in particular ubiquitination is clearly enriched, in particular in the combined cluster with down-regulated expression pattern (4, 5, and 6). It may make sense that proteins of down-regulated genes are ubiquitinated, in order to speed up the process of down-regulation. It is also interesting that there is a clear depletion of DNA-binding in genes with a stable (housekeeping-like) expression pattern. It is possible that these transcription factors rely on interaction with open chromatin initiated by other transcription factors, and are therefore less actively regulated than such key factors.

**Table 7:** **Results for TF expression changes during cell differentiation**

Category*	**Term**	**Observed**	**Expected**	**Pvalue**	**Benjamini**	**MCC**
1	Ubiquitination	4	1	4.20E-02	3.36E-01	0.049
	Sumoylation	2	0	4.84E-02	1.93E-01	0.062
6	O-GlcNAc	3	0	9.69E-03	7.75E-02	0.086
	Ubiquitination	16	9	1.58E-02	6.31E-02	0.058
	Methylation	9	4	2.35E-02	6.27E-02	0.059
8	Ubiquitination	17	9	1.36E-03	1.09E-02	0.074
	PPI	10	5	2.39E-02	9.58E-02	0.070
1,2,3	PPI	9	4	1.57E-02	1.26E-01	0.072
4,5,6	Ubiquitination	43	29	1.48E-03	1.18E-02	0.074
	Methylation	21	12	1.03E-02	4.11E-02	0.061
	Sumoylation	12	6	2.98E-02	7.93E-02	0.054
	O-GlcNAc	4	1	4.53E-02	9.07E-02	0.052
7,8,9,10	DNA_Binding	28	38	7.49E-03	5.99E-02	−0.062
	Ubiquitination	38	28	1.32E-02	5.29E-02	0.058

*Indicates TFs with similar expression profiles: 1, 2, 3 - Up-regulated; 4, 5, 6 - Down-regulated; 7, 8, 9, 10 - No clear change.

A paper by Lawrence *et al.* identified somatic point mutations in exome sequences from 4742 human cancers with matched normal-tissue samples across 21 cancer types [35]. Frequently mutated genes were identified and analyzed according to whether the gene was mainly mutated in a single cancer, or across many cancers. This made it possible to identify subsets of genes, here identified as gene set I (mainly mutated across many cancers), II (highly mutated in a few cancers), and III (highly mutated across many cancers). The last set could further be divided into IIIA and IIIB, where B consists of the genes that are most broadly mutated [see Additional file 7]. The analysis shows that many features are enriched, but often represented by a small number of genes (Table 8, full results in Table S5 [see Additional file 2]). The most significant enrichments are for PTMs. However, it is possible that this is influenced by experimental bias, as known cancer genes may have been more frequently tested for PTMs. We also see that DNA-binding again is depleted, possibly indicating that TFs with a strong and easily identified DBD are more essential to cellular function, and therefore less frequently mutated. Also some Pfam domains show a small enrichment, in particular for the SET and PHD domains. These domains are found frequently for example in members of the MLL family, which catalyze H3K4 methylation as part of a large multiprotein complex containing several chromatin remodeling factors. More than 70% of infant leukemia and approximately 10% of adult human leukemia display chromosomal translocations of the MLL (KMT2A) gene, and 450 functionally diverse MLL fusions having been identified. However, it is interesting that in all fusion proteins the C-terminal SET domain is lost and consequently they lack H3K4 methyltransferase activity [36]. The PLU-1/JARID1B is a nuclear protein which is expressed in a high proportion of breast cancers. Two PHD domains in PLU-1/JARID1B are involved in transcriptional repression. Indeed the interaction between the class II HDACs (histone deacetylase) and PLU-1/JARID1B depends on functional PHD domains, and is responsible for transcriptional repression [37].

**Table 8:** **Selected results for TFs that are frequently mutated in cancer**

Category*	**Term**	**Observed**	**Expected**	**Pvalue**	**Benjamini**	**MCC**
II + IIIAB	Acetylation	48	26	1.823E-08	1.46E-07	0.125
	Ubiquitination	47	27	2.08E-07	8.31E-07	0.118
	Methylation	26	11	1.21E-05	3.23E-05	0.110
	PF00856(SET)	6	0	1.21E-05	8.80E-03	0.164
	PF13771(zf-HC5HC2H)	4	0	4.90E-05	1.78E-02	0.175
	PF00628(PHD)	8	1	1.78E-04	2.58E-02	0.114
	Sumoylation	14	5	1.14E-03	2.28E-0	0.082
	O-GlcNAc	5	1	7.03E-03	1.12E-02	0.078
II	Acetylation	21	12	1.67E-03	1.33E-02	0.073
	Ubiquitination	20	12	6.61E-03	2.64E-02	0.063
	Methylation	11	5	1.23E-02	3.28E-02	0.062
	O-GlcNAc	3	0	1.89E-02	3.78E-02	0.073
IIIB	Sumoylation	5	1	3.51E-03	2.80E-02	0.085
I + IIIAB	Ubiquitination	32	16	2.69E-07	2.15E-06	0.114
	Acetylation	30	16	6.44E-06	2.12E-05	0.101
	Methylation	19	7	7.94E-06	2.12E-05	0.114
	PF00856(SET)	5	0	1.67E-05	5.78E-03	0.178
	PF00628(PHD)	7	1	4.70E-05	8.56E-03	0.136
	PF13771(zf-HC5HC2H)	3	0	3.17E-04	2.25E-02	0.168
	Sumoylation	10	3	1.82E-03	3.64E-03	0.082
	DNA_Binding	15	22	9.56E-03	1.53E-02	−0.061
IIIAB	Acetylation	27	14	5.47E-06	2.56E-05	0.102
	Ubiquitination	27	14	6.39E-06	2.56E-05	0.101
	PF00628(PHD)	6	0	1.82E-04	2.64E-02	0.125
	PF00439(Bromodomain)	4	0	4.78E-04	4.35E-02	0.132
	Methylation	15	6	2.79E-04	7.44E-04	0.091
	Sumoylation	9	3	2.28E-03	4.56E-03	0.081
	DNA_Binding	12	19	5.45E-03	8.71E-03	−0.065
I	Methylation	4	0	5.47E-03	4.38E-02	0.078

*Indicates TFs with similar mutation profiles: I - Mainly mutated across many cancers; II - Highly mutated in a few cancers; IIIA - Highly mutated across many cancers; IIIB - Even more highly mutated across many cancers.

Vaquerizas *et al.* [16] have published an analysis of 1391 manually curated sequence-specific DNA-binding transcription factors. They looked into the tissue distribution of TF expression, and identified a bi-modal distribution; 37% of the TFs showed significant expression in at least one tissue, 32% of these were expressed in most tissues, whereas the majority was expressed only in a subset (typically 1–3 tissues). We used these three subsets (general tissue distribution, specific distribution, and unknown; [see Additional file 8]) as input for analysis. The results are shown in Table 9 (full results in Table S6 [see Additional file 2]). They show an expected enrichment for DNA-binding, since this particular dataset has been selected for DNA-binding TFs. They also show a depletion of PTMs and PPIs in the set with unknown tissue distribution. This most likely indicates the same problem as before with respect to data bias; many of these TFs have been less studied, and the lack of PTMs most likely reflects a lack of experimental data, and not that they are less frequently modified. It is probably more relevant that the tissue-specific TFs are more likely to be sumoylated or be hormone receptors than the general ones, as this may reflect mechanisms for tissue-specific regulation (see e.g. [38]). It is also interesting that the KRAB domain is depleted in the tissue-specific set, but enriched in the unknown (not expressed) set, as KRAB is a known transcriptional repressor domain [39].

**Table 9:** **Selected results for TFs with differences in tissue specificity**

Category*	**Term**	**Observed**	**Expected**	**Pvalue**	**Benjamini**	**MCC**
General	DNA_Binding	126	85	1.36E-10	1.09E-09	0.166
Specific	DNA_Binding	306	205	1.92E-10	1.53E-09	0.280
	Sumoylation	57	31	1.85E-06	7.39E-06	0.115
	PF00104(Hormone_recep)	28	7	2.32E-11	1.69E-08	0.179
	PF01352(KRAB)	16	40	9.20E-07	1.67E-04	−0.103
Unknown	DNA_Binding	702	486	2.82E-10	2.26E-09	0.459
	Ubiquitination	229	355	3.19E-10	1.28E-09	−0.263
	Methylation	105	149	1.68E-07	4.47E-07	−0.116
	PPI	146	172	1.48E-03	2.37E-03	−0.091
	PF01352(KRAB)	200	96	2.11E-10	7.66E-08	0.324

*Indicates TFs found in many tissues (general), a few tissues (specific), or unknown (due to very low or no expression).

## Conclusions

A combination of literature-based curation and prediction methods has been used to build a comprehensive list of transcription factor properties, and this list has been applied towards investigating relationships between TF properties, TF–TF (protein–protein) interactions, and external data, and used to find significant correlations and enriched or depleted features. The results show that the comprehensive list is a useful data analysis resource for researchers working on gene regulation. However, it also shows that such analyses are easily biased by incomplete data or by how the gene sets have been selected. This mirrors to some extent the recent results by Rolland *et al.* [40], where they identified a strong bias in existing PPI data towards well-studied proteins.

### Availability of supporting data

The data sets supporting the results of this article are included within the article and its additional files, or were downloaded from open sources as shown in Methods.

## Additional files

Additional file 1:
**Manually checked Pfam domains for DNA-binding.**
Additional file 2: Figure S1. Distribution of Pfam domain types. Domains with less than 5 occurrences are grouped under “Others”. **Figure S2.** Plot of data used to predict new DNA-binding domain types. Only domains that overlap with DBD-Threader predictions are shown. The classification line for SVM-based classification with a linear kernel is indicated. **Figure S3.** Enriched PPI domain pairs. **Table S1.** Selected enriched terms according to GOrilla. **Table S2.** Selected enriched terms according to DAVID. **Table S3.** Associations between property-based subgroups. **Table S4.** Output from enrichment analysis of data from Tuomela et al. **Table S5.** Output from enrichment analysis of data from Lawrence et al. **Table S6.** Output from enrichment analysis of data from Vaquerizas et al. Additional file 3:
**Main table of transcription factor properties.**
Additional file 4:
**Enriched domains and domain pairs in PPI.**
Additional file 5:
**Software for data analysis.**
Additional file 6:
**Data from Tuomela et al. for Table S4.**
Additional file 7:
**Data from Lawrence** ***et al.*** **for Table S5.**
Additional file 8:
**Data from Vaquerizas** ***et al.*** **for Table S6.**

## Abbreviations

TF: Transcription factor
DBD: DNA-binding domain
PPI: Protein–protein interaction
TFBS: Transcription factor binding site
PTM: Post-translational modifications
PWM: Position weight matrix
PFM: Position frequency matrix
HMM: Hidden Markov model
FN: False negative
FP: False positive
TP: True positive
TN: True negative
Sn: Sensitivity
Sp: Specificity
PPV: Positive predictive value
MCC: Matthews correlation coefficient
Bp: Base pair
SVM: Support vector machine
