# Supplementary material for: A property-based analysis of human transcription factors
Source: BMC Res Notes. 2015 Mar 14;8:82. doi: 10.1186/s13104-015-1039-6 (PMC4373352; doi:10.1186/s13104-015-1039-6)
Supplement: Additional file 2: Figure S1. — Distribution of Pfam domain types. Domains with less than 5 occurrences are grouped under “Others”. Figure S2. Plot of data used to predict new DNA-binding domain types. Only domains that overlap with DBD-Threader predictions are shown. The classification line for SVM-based classification with a linear kernel is indicated. Figure S3. Enriched PPI domain pairs. Table S1. Selected enriched terms according to GOrilla. Table S2. Selected enriched terms according to DAVID. Table S3. Associations between property-based subgroups. Table S4. Output from enrichment analysis of data from Tuomela et al. Table S5. Output from enrichment analysis of data from Lawrence et al. Table S6. Output from enrichment analysis of data from Vaquerizas et al. [file 13104_2015_1039_MOESM2_ESM.pdf]

Additional file 2

Figure S1 - Distribution of Pfam domain types. Domains with less than 5 occurrences are grouped under “Others”.

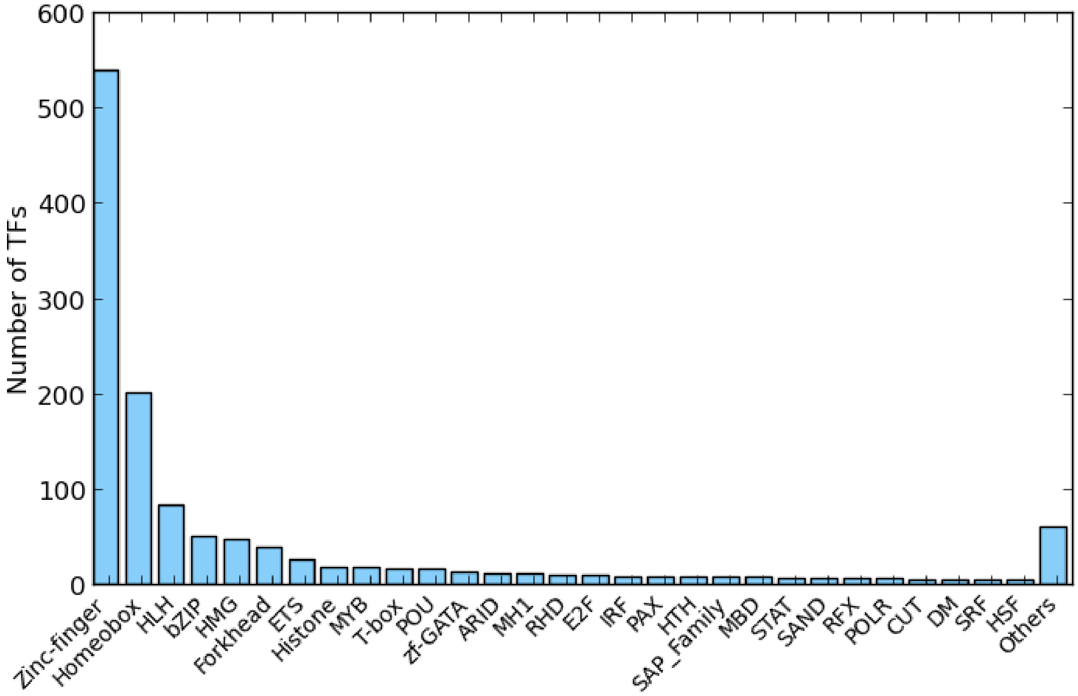

Figure S2 - Plot of data used to predict new DNA-binding domain types. Only domains that overlap with DBD-Threader predictions are shown. The classification line for SVM-based classification with a linear kernel is indicated.

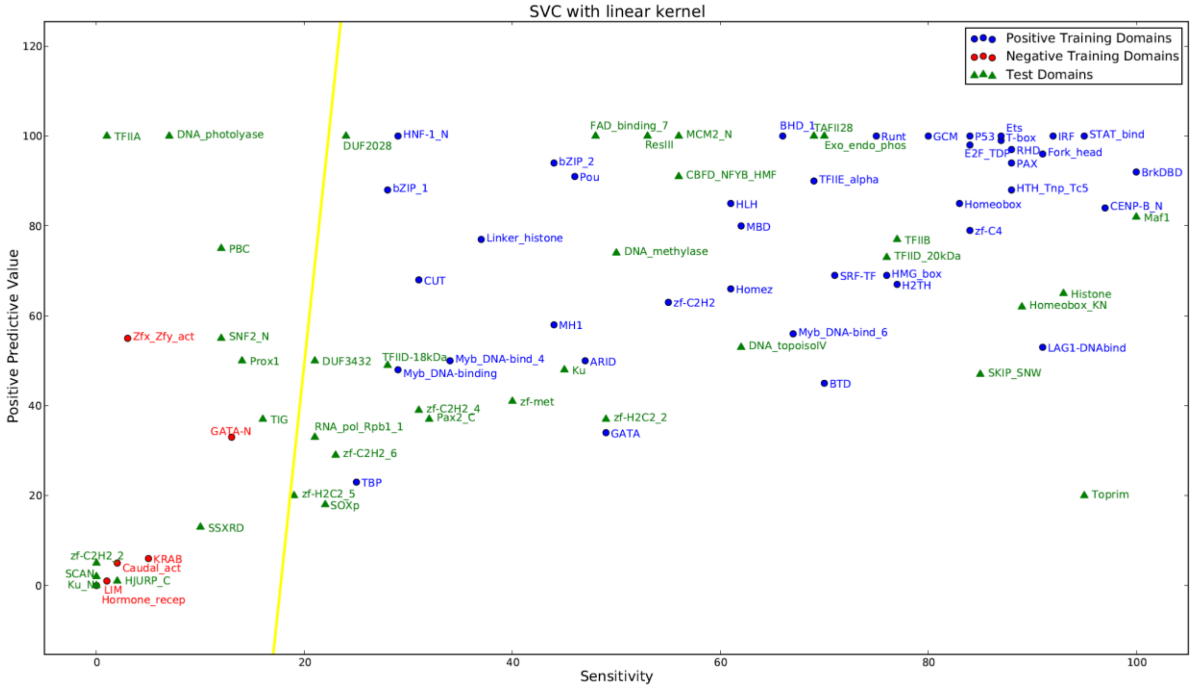

Figure S3 - Enriched PPI domain pairs

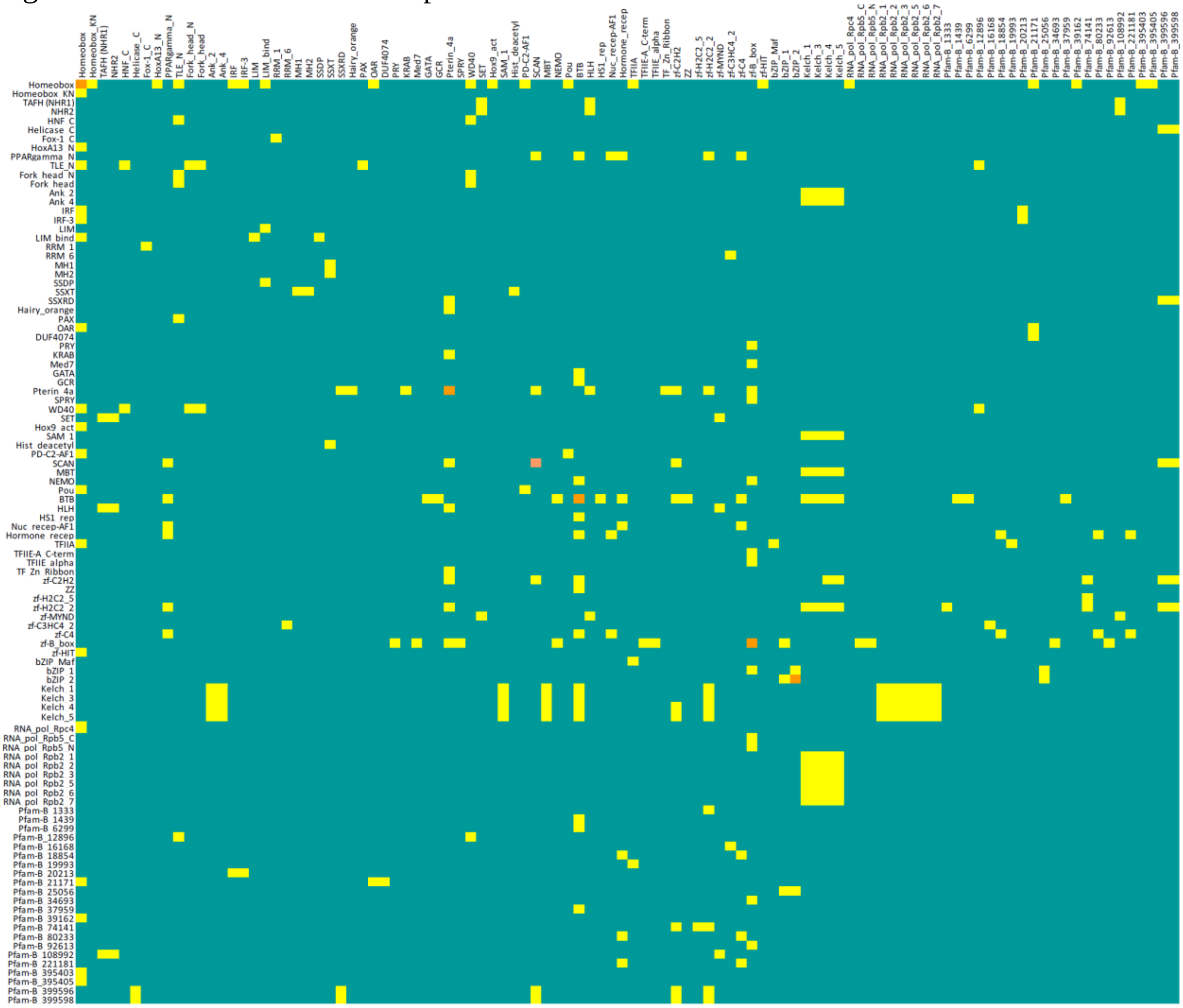

Table S1 - Selected enriched terms according to GOrilla

Description	P-value	FDR q-value	Enrichment (N, B, n, b)
**DNA_Binding**			
DNA binding	2.11E-185	1.72E-182	1.28 (1939,1475,1206,1174)
RNA polymerase II core promoter proximal region sequence-specific DNA binding	6.34E-16	2.26E-14	1.46 (1939,148,1206,134)
protein dimerization activity	4E-8	1.13E-6	1.24 (1939,254,1206,196)
ligand-activated sequence-specific DNA binding RNA polymerase II transcription factor activity	1.61E-7	4.41E-6	1.54 (1939,45,1206,43)
direct ligand regulated sequence-specific DNA binding transcription factor activity	1.61E-7	4.26E-6	1.54 (1939,45,1206,43)
RNA polymerase II core promoter proximal region sequence-specific DNA binding transcription factor activity involved in negative regulation of transcription	7.9E-7	2.02E-5	1.46 (1939,56,1206,51)
signaling receptor activity	3E-6	7.46E-5	1.44 (1939,57,1206,51)
protein heterodimerization activity	1.68E-5	4.05E-4	1.27 (1939,129,1206,102)
E-box binding	7E-5	1.64E-3	1.50 (1939,31,1206,29)
core promoter sequence-specific DNA binding	7.87E-5	1.79E-3	1.37 (1939,60,1206,51)
HMG box domain binding	4.83E-4	1.07E-2	1.61 (1939,16,1206,16)
steroid binding	7.8E-4	1.56E-2	1.61 (1939,15,1206,15)
**Non_DNA_Binding**			
catalytic activity	1.07E-49	8.75E-47	2.01 (1939,305,735,232)
RNA binding	3.95E-34	1.62E-31	2.00 (1939,222,735,168)
nucleoside phosphate binding	1.17E-26	2.4E-24	2.11 (1939,144,735,115)
protein binding	1.66E-19	1.7E-17	1.22 (1939,1135,735,524)
transcription cofactor activity	9.56E-12	4.61E-10	1.42 (1939,359,735,193)
histone binding	1.03E-10	3.39E-9	2.07 (1939,60,735,47)
ubiquitin-protein transferase activity	2.29E-10	7.21E-9	2.40 (1939,33,735,30)
methylated histone binding	3.8E-10	1.11E-8	2.54 (1939,26,735,25)
protein binding transcription factor activity	3.61E-9	8.69E-8	1.34 (1939,389,735,198)
transcription coactivator activity	1.87E-7	3.93E-6	1.44 (1939,207,735,113)
hormone receptor binding	8.15E-6	1.26E-4	1.64 (1939,77,735,48)
histone deacetylase activity (H4-K16 specific)	8.32E-6	1.24E-4	2.64 (1939,12,735,12)
histone deacetylase activity (H3-K9 specific)	2.22E-5	3.18E-4	2.64 (1939,11,735,11)
deacetylase activity	2.51E-5	3.12E-4	2.33 (1939,17,735,15)
**Acetylation**			
protein binding	8.76E-13	2.39E-10	1.15 (1939,1133,879,590)
nucleoside phosphate binding	1.06E-11	2.18E-9	1.59 (1939,144,879,104)
transcription factor binding	2.12E-6	2.17E-4	1.28 (1939,292,879,169)
chromatin binding	2.14E-5	7.63E-4	1.26 (1939,264,879,151)
structure-specific DNA binding	2.27E-5	7.76E-4	1.38 (1939,136,879,85)
histone binding	7.76E-5	1.99E-3	1.54 (1939,60,879,42)
chromatin DNA binding	1.44E-4	3.47E-3	1.55 (1939,54,879,38)
core promoter binding	3.54E-4	7.83E-3	1.39 (1939,92,879,58)
carbon-oxygen lyase activity	7.91E-4	1.54E-2	2.21 (1939,9,879,9)
histone acetyltransferase activity	9.83E-4	1.87E-2	1.64 (1939,31,879,23)
**Non_Acetylation**			
sequence-specific DNA binding transcription factor activity	1.36E-6	1.11E-3	1.11 (1939,887,1061,537)
nucleic acid binding transcription factor activity	1.74E-6	7.14E-4	1.11 (1939,888,1061,537)
sequence-specific DNA binding	5.9E-5	1.61E-2	1.12 (1939,620,1061,379)
**Methylation**			
nucleoside phosphate binding	5.75E-15	1.57E-12	2.43 (1939,144,372,67)
protein binding	2.67E-8	3.12E-6	1.21 (1939,1135,372,264)
transferase activity	3.03E-7	2.07E-5	1.82 (1939,169,372,59)
chromatin binding	3.93E-7	2.48E-5	1.62 (1939,264,372,82)
transcription coactivator activity	1.98E-4	4.16E-3	1.51 (1939,207,372,60)
hormone receptor binding	2.33E-4	4.65E-3	1.90 (1939,77,372,28)
receptor binding	2.35E-4	4.59E-3	1.72 (1939,112,372,37)
structure-specific DNA binding	9.56E-4	1.63E-2	1.57 (1939,136,372,41)
histone methyltransferase activity	9.69E-4	1.62E-2	2.50 (1939,25,372,12)
**Non_Methylation**			
nucleic acid binding transcription factor activity	5.56E-12	4.55E-9	1.08 (1939,888,1567,776)
sequence-specific DNA binding transcription factor activity	6.47E-12	2.65E-9	1.08 (1939,887,1567,775)
sequence-specific DNA binding RNA polymerase II transcription factor activity	7.68E-7	2.1E-4	1.11 (1939,348,1567,312)
sequence-specific DNA binding	1.93E-6	3.94E-4	1.07 (1939,620,1567,538)
RNA polymerase II core promoter proximal region sequence-specific DNA binding transcription factor activity	5.09E-5	8.33E-3	1.13 (1939,188,1567,171)
RNA polymerase II transcription regulatory region sequence-specific DNA binding transcription factor activity involved in positive regulation of transcription	8.71E-5	1.19E-2	1.12 (1939,184,1567,167)
RNA polymerase II core promoter proximal region sequence-specific DNA binding transcription factor activity involved in positive regulation of transcription	2.43E-4	2.84E-2	1.13 (1939,141,1567,129)

**O_GlcNAc**			
enzyme binding	1.21E-8	9.88E-6	4.39 (1939,194,41,18)
protein binding	6.83E-6	2.8E-3	1.54 (1939,1133,41,37)
histone deacetylase binding	2.71E-4	7.41E-2	6.31 (1939,45,41,6)
protein phosphatase 2A binding	4.36E-4	8.94E-2	47.29 (1939,2,41,2)
protein phosphatase binding	4.57E-4	7.48E-2	17.73 (1939,8,41,3)
**Phosphorylation**			
ion binding	4.52E-5	3.7E-2	1.03 (1939,923,1782,872)
protein binding	4.93E-5	2.02E-2	1.02 (1939,1133,1782,1065)
cation binding	2.84E-4	7.77E-2	1.03 (1939,862,1782,813)
metal ion binding	2.84E-4	5.82E-2	1.03 (1939,862,1782,813)
enzyme binding	7.9E-4	1.29E-1	1.06 (1939,194,1782,189)
**PTM**			
protein binding	3.12E-6	2.55E-3	1.02 (1939,1135,1827,1093)
**Sumoylation**			
protein binding	1.32E-19	5.4E-17	1.49 (1939,1133,189,165)
sequence-specific DNA binding	3E-12	4.1E-10	1.73 (1939,617,189,104)
transcription factor binding	6.73E-10	6.12E-8	2.11 (1939,292,189,60)
RNA polymerase II regulatory region sequence-specific DNA binding	4.12E-9	3.38E-7	2.16 (1939,252,189,53)
RNA polymerase II core promoter proximal region sequence-specific DNA binding transcription factor activity	3.59E-8	2.45E-6	2.30 (1939,187,189,42)
sequence-specific DNA binding RNA polymerase II transcription factor activity	8.63E-8	4.71E-6	1.85 (1939,344,189,62)
core promoter binding	1.86E-7	8.03E-6	2.90 (1939,92,189,26)
chromatin binding	1.92E-7	7.86E-6	1.98 (1939,264,189,51)
transcription factor binding transcription factor activity	7.03E-6	2.3E-4	1.65 (1939,386,189,62)
protein binding transcription factor activity	9.26E-6	2.92E-4	1.64 (1939,389,189,62)
ubiquitin protein ligase binding	1.03E-5	2.82E-4	3.59 (1939,40,189,14)
protein dimerization activity	1.57E-5	3.88E-4	1.82 (1939,254,189,45)
receptor binding	3.02E-5	6.34E-4	2.31 (1939,111,189,25)
transcription cofactor activity	1.24E-4	2.11E-3	1.57 (1939,359,189,55)
receptor activity	1.96E-4	3.28E-3	2.53 (1939,69,189,17)
transcription coactivator activity	4.49E-4	6.82E-3	1.73 (1939,207,189,35)
**Ubiquitination**			
protein binding	3.71E-30	3.04E-27	1.24 (1939,1133,888,641)
nucleoside phosphate binding	2.01E-20	3.29E-18	1.79 (1939,144,888,118)
transferase activity	7.29E-15	2.98E-13	1.62 (1939,169,888,125)
transcription cofactor activity	3.27E-8	8.12E-7	1.28 (1939,359,888,211)
protein binding transcription factor activity	2.31E-7	5.39E-6	1.25 (1939,389,888,223)
transcription coactivator activity	6.99E-7	1.59E-5	1.35 (1939,207,888,128)
transcription factor binding	2.64E-6	5.41E-5	1.27 (1939,292,888,170)
receptor binding	1.25E-4	1.89E-3	1.38 (1939,111,888,70)
chromatin binding	2.09E-4	3.11E-3	1.22 (1939,264,888,148)
histone binding	2.92E-4	4.12E-3	1.49 (1939,60,888,41)
hormone receptor binding	4.4E-4	5.91E-3	1.42 (1939,77,888,50)
**Non_Ubiquitination**			
DNA binding	6.99E-14	5.73E-11	1.09 (1939,1473,1052,869)
nucleic acid binding transcription factor activity	3.55E-12	1.45E-9	1.16 (1939,888,1052,557)
sequence-specific DNA binding transcription factor activity	4.74E-12	1.29E-9	1.16 (1939,887,1052,556)
nucleic acid binding	7.02E-8	1.44E-5	1.05 (1939,1599,1052,912)
heterocyclic compound binding	1.21E-7	1.98E-5	1.05 (1939,1617,1052,920)
organic cyclic compound binding	1.21E-7	1.65E-5	1.05 (1939,1617,1052,920)
cation binding	8.48E-6	9.93E-4	1.10 (1939,862,1052,515)
metal ion binding	8.48E-6	8.69E-4	1.10 (1939,862,1052,515)
sequence-specific DNA binding	1.82E-5	1.65E-3	1.13 (1939,620,1052,379)
sequence-specific DNA binding RNA polymerase II transcription factor activity	1.95E-4	1.6E-2	1.16 (1939,348,1052,219)
**PPI**			
protein binding	6.83E-5	4.79E-2	1.11 (1203,740,475,324)
transcription factor binding	1.38E-4	4.83E-2	1.31 (1203,185,475,96)
protein domain specific binding 8.34E-4	8.34E-4	1.95E-1	1.44 (1203,81,475,46)

Table S2 - Selected enriched terms according to DAVID

SubClass	Category	Term	Count	%	Pvalue	Benjamini
DNA_binding						
	PFAM	zf-C2H2	485	40.3	4.0E-77	3.3E-74
	PFAM	KRAB	240	19.9	7.4E-37	3.1E-34
	PFAM	KRAB box	218	18.1	2.5E-33	6.7E-31
	PFAM	Homeobox	193	16.0	2.5E-31	5.1E-29
	PFAM	Homeobox domain	161	13.4	5.7E-26	9.3E-24
	PFAM	zf-C4	44	3.7	8.5E-8	1.2E-5
	PFAM	Helix-loop-helix DNA-binding domain	62	5.1	9.9E-8	1.2E-5
	PFAM	Zinc finger, C4 type	43	3.6	1.3E-7	1.3E-5
	PFAM	HLH	81	6.7	2.5E-7	2.3E-5
	PFAM	Fork_head	39	3.2	7.2E-7	5.9E-5
	PFAM	Fork head domain	38	3.2	1.1E-6	8.2E-5
	PFAM	HMG	42	3.5	1.6E-6	1.1E-4
	PFAM	HMG_box	44	3.7	4.2E-6	2.5E-4
	PFAM	Hormone_recep	38	2.8	4.3E-5	4.2E-2
	PFAM	Ligand-binding domain of nuclear hormone receptor	43	3.6	6.2E-6	3.4E-4
	PFAM	SCAN	45	3.7	1.3E-5	6.7E-4
	PFAM	SCAN domain	45	3.7	1.3E-5	6.7E-4
	PFAM	bZIP_1	29	2.4	4.7E-5	2.3E-3
	PFAM	BTB	43	3.6	2.9E-4	1.3E-2
	GOTERM_MF_FAT	DNA binding	1174	97.5	1.7E-130	5.1E-128
	GOTERM_MF_FAT	transcription factor activity	723	60.0	7.3E-63	7.6E-61
	GOTERM_MF_FAT	specific RNA polymerase II transcription factor activity	39	3.2	5.8E-4	1.6E-2
Non_DNA_binding						
	SP_COMMENT_TYPE	PTM	237	33.2	1.6E-12	1.3E-11
	SP_PIR_KEYWORDS	phosphoprotein	472	66.1	2.1E-29	5.8E-27
	SP_PIR_KEYWORDS	acetylation	208	29.1	3.7E-23	3.5E-21
	SP_PIR_KEYWORDS	chromatin regulator	82	11.5	4.7E-11	1.1E-9
	PFAM	Ank	52	7.3	1.8E-20	1.6E-17
	PFAM	zf-C3HC4	27	3.8	2.3E-11	1.1E-8
	PFAM	RRM_1	30	4.2	4.3E-10	1.3E-7
	PFAM	Zinc finger, C3HC4 type	23	3.2 9	1.4E-9	3.2E-7
	PFAM	PHD	49	6.9	8.2E-9	1.5E-6
	PFAM	RBD	24	3.4	2.4E-8	3.7E-6
	PFAM	WD40	19	2.7	5.4E-7	7.0E-5
	PFAM	PHD-finger	25	3.5	4.6E-6	5.2E-4
	PFAM	zf-B_box	13	1.8	3.1E-5	3.2E-3
	PFAM	SOCS_box	11	1.5	2.2E-4	2.0E-2
	PFAM	KH_1	11	1.5	2.2E-4	2.0E-2
	PFAM	SOCS box	11	1.5	2.2E-4	2.0E-2
	PFAM	Hist_deacetyl	10	1.4	5.7E-4	4.6E-2
	PFAM	zf-DHHC	10	1.4	5.7E-4	4.6E-2
	PFAM	Histone deacetylase domain	10	1.4	5.7E-4	4.6E-2
	GOTERM_MF_FAT	transcription cofactor activity	168	23.5	9.5E-20	1.2E-17
	GOTERM_MF_FAT	transcription factor binding	197	27.6	2.4E-16	2.2E-14
	GOTERM_BP_FAT	chromatin modification	104	14.6	1.4E-17	7.3E-15
	GOTERM_MF_FAT	hormone receptor binding	43	6.0	4.1E-12	2.0E-10
	GOTERM_BP_FAT	histone modification	55	7.7	7.0E-16	2.0E-13
	GOTERM_BP_FAT	chromatin organization	113	15.8	4.2E-12	5.9E-10
	GOTERM_MF_FAT	protein ubiquitination	17	2.4	3.2E-6	1.8E-4
Non_PPI						
	PFAM	KRAB	116	16.2	4.4E-8	3.3E-5
	PFAM	KRAB box	108	15.1	1.5E-7	5.6E-5
	PFAM	zf-C2H2	203	28.4	4.0E-6	1.0E-3
PTM						
	SP_PIR_KEYWORDS	phosphoprotein	927	51.5	5.3E-12	1.9E-9
Non_PTM						
	PFAM	Homeobox	28	23.3	4.7E-5	7.5E-3
	UP_SEQ_FEATURE	DNA-binding region:Homeobox	25	20.8	2.9E-5	9.6E-3
Phosphorylation						
	SP_COMMENT_TYPE	PTM	440	25.1	2.3E-3	1.4E-2
	SP_PIR_KEYWORDS	phosphoprotein	925	52.7	2.6E-21	9.1E-19
Non_Phosphorylation						
	PFAM	Homeobox	37	22.4	5.4E-6	1.1E-3
	PFAM	Homeobox domain	29	17.6	2.6E-4	2.7E-2
	GOTERM_MF_FAT	sequence-specific DNA binding	63	38.0	2.3E-4	2.7E-2
Acetylation						
	SP_COMMENT_TYPE	PTM	291	34.2	4.9E-20	1.2E-18

	PFAM	PHD	51	6.0	2.8E-6	3.1E-3
	PFAM	Bromodomain	23	2.7	7.9E-5	4.3E-2
	SP_PIR_KEYWORDS	acetylation	293	34.5	2.4E-69	7.0E-67
	SP_PIR_KEYWORDS	phosphoprotein	546	64.2	6.4E-31	9.5E-29
	GOTERM_BP_FAT	chromosome organization	144	16.9	3.7E-16	8.5E-13
	GOTERM_BP_FAT	chromatin organization	137	16.1	1.6E-14	2.1E-11
	GOTERM_BP_FAT	chromatin modification	103	12.1	8.1E-9	6.9E-6
	SP_PIR_KEYWORDS	methylation	29	3.4	9.0E-5	2.7E-3
	GOTERM_BP_FAT	histone acetylation	27	3.2	1.4E-4	2.1E-2
	GOTERM_BP_FAT	chromatin remodeling	29	3.4	4.8E-4	6.3E-2
Non_Acetylation						
	PFAM	Homeobox	158	14.8	1.9E-13	1.8E-10
	PFAM	Homeobox domain	132	12.3	2.3E-11	1.1E-8
	GOTERM_MF_FAT	transcription factor activity	528	49.3	1.6E-7	5.3E-5
	GOTERM_MF_FAT	sequence-specific DNA binding	330	30.8	1.8E-7	2.8E-5
Methylation						
	SP_COMMENT_TYPE	PTM	127	36.2	1.7E-8	2.1E-7
	PFAM	SNF2_N	13	3.7	2.8E-5	1.7E-2
	PFAM	AT_hook	12	3.4	4.9E-5	1.5E-2
	PFAM	PHD	27	7.7	7.4E-5	1.5E-2
	PFAM	KH_1	9	2.6	9.6E-5	1.4E-2
	PFAM	RBD	14	4.0	1.7E-4	2.0E-2
	PFAM	Helicase_C	13	3.7	1.8E-4	1.8E-2
	PFAM	RRM_1	16	4.6	2.0E-4	1.7E-2
	PFAM	PHD-finger	15	4.3	5.4E-4	4.0E-2
	PFAM	Helicase conserved C-terminal domain	11	3.1	5.8E-4	3.8E-2
	PFAM	linker histone H1 and H5 family	7	2.0	6.3E-4	3.8E-2
	PFAM	Linker_histone 7	7	2.0	6.3E-4	3.8E-2
	SP_PIR_KEYWORDS	phosphoprotein	259	73.8	1.8E-24	3.9E-22
	SP_PIR_KEYWORDS	methylation	30	8.5	1.3E-15	1.4E-13
	SP_PIR_KEYWORDS	acetylation	115	32.8	2.4E-14	1.7E-12
	GOTERM_BP_FAT	chromosome organization	75	21.4	1.3E-11	2.0E-8
	GOTERM_BP_FAT	chromatin organization	71	20.2	1.6E-10	1.2E-7
	GOTERM_BP_FAT	chromatin modification	54	15.4	2.5E-7	9.7E-5
	GOTERM_MF_FAT	histone methyltransferase activity	13	3.7	7.4E-5	3.0E-3
	GOTERM_MF_FAT	histone acetyltransferase activity	13	3.7	2.4E-4	7.4E-3
	GOTERM_MF_FAT	transcription coactivator activity	49	14.0	6.2E-4	1.6E-2
	GOTERM_MF_FAT	transcription factor binding	90	25.6	1.6E-3	3.1E-2
Non_Methylation						
	PFAM	Homeobox	189	12.1	1.9E-8	2.6E-5
	PFAM	Homeobox domain	157	10.0	9.4E-7	6.4E-4
	PFAM	KRAB box	210	13.4	1.9E-6	8.8E-4
	PFAM	KRAB	230	14.7	2.0E-6	6.7E-4
	PFAM	zf-C2H2	448	28.6	5.2E-5	1.4E-2
	GOTERM_MF_FAT	transcription factor activity	741	47.2	3.4E-9	1.5E-6
	GOTERM_MF_FAT	sequence-specific DNA binding	451	28.8	2.7E-8	6.0E-6
	GOTERM_BP_FAT	transcription regulator activity	1003	64.0	4.2E-3	3.7E-1
	SP_PIR_KEYWORDS	protein dimerization activity	134	8.5	5.3E-3	3.8E-1
Ubiquitination						
	SP_COMMENT_TYPE	PTM	298	34.4	1.8E-21	1.4E-20
	PFAM	SNF2_N	20	2.3	2.3E-6	2.6E-3
	PFAM	Helicase_C	21	2.4	3.0E-5	1.7E-2
	PFAM	Bromodomain	23	2.7	8.8E-5	3.3E-2
	SP_PIR_KEYWORDS	phosphoprotein	578	66.7	3.5E-43	1.1E-40
	SP_PIR_KEYWORDS	acetylation	261	30.1	5.7E-39	8.6E-37
	GOTERM_BP_FAT	chromosome organization	144	16.6	1.8E-15	4.4E-12
	GOTERM_BP_FAT	chromatin organization	138	15.9	2.1E-14	1.8E-11
	GOTERM_MF_FAT	transcription factor binding	232	26.8	3.2E-13	2.8E-11
	GOTERM_MF_FAT	transcription coactivator activity	116	13.4	7.0E-10	4.4E-8 4
	SP_PIR_KEYWORDS	chromatin regulator	89	10.3	1.6E-9	6.0E-8
	SP_PIR_KEYWORDS	methylation	31	3.6	6.6E-6	1.3E-4
	GOTERM_MF_FAT	transcription activator activity	186	21.5	3.3E-5	9.5E-4
	GOTERM_BP_FAT	chromatin remodeling	28	3.2	2.0E-3	8.5E-2
Non_Ubiquitination						
	PFAM	zf-C2H2	369	35.0	5.3E-20	4.6E-17
	PFAM	KRAB	197	18.7	1.3E-16	4.8E-14
	PFAM	KRAB box	177	16.8	6.2E-14	1.8E-11
	PFAM	Homeobox	158	15.0	1.1E-13	2.3E-11
	PFAM	Homeobox domain	131	12.4	4.9E-11	8.4E-9
	PFAM	Fork_head	36	3.4	3.2E-6	4.6E-4
	PFAM	Fork head domain	35	3.3	5.3E-6	6.6E-4

	SP_PIR_KEYWORDS	dna-binding	809	76.8	2.3E-21	5.0E-19
	GOTERM_MF_FAT	DNA binding	881	83.6	6.6E-14	2.0E-11
	GOTERM_BP_FAT	regulation of transcription	943	89.5	4.4E-9	3.6E-6
	GOTERM_MF_FAT	transcription factor activity	525	49.8	8.3E-9	1.2E-6
	SP_PIR_KEYWORDS	transcription regulation	841	79.8	6.6E-6	2.1E-4
Sumoylation						
	SP_COMMENT_TYPE	PTM	106	56.7	5.8E-24	1.3E-22
	PFAM	zf-C4	17	9.1	1.3E-6	4.4E-4
	PFAM	Hormone_recep	17	9.1	2.6E-6	4.4E-4
	PFAM	Zinc finger, C4 type	16	8.6	5.2E-6	6.0E-4
	PFAM	Ligand-binding domain of nuclear hormone receptor	16	8.6	9.8E-6	8.5E-4
	PFAM	SRF-TF	5	2.7	4.1E-4	2.8E-2
	PFAM	SRF-type transcription factor	5	2.7	4.1E-4	2.8E-2
	SP_PIR_KEYWORDS	phosphoprotein	144	77.0	9.1E-16	5.3E-14
	SP_PIR_KEYWORDS	activator	71	38.0	3.7E-8	1.7E-6
	GOTERM_MF_FAT	sequence-specific DNA binding	83	44.4	1.2E-7	1.5E-5
	GOTERM_MF_FAT	transcription activator activity	62	33.2	1.9E-7	1.6E-5
	SP_PIR_KEYWORDS	acetylation	61	32.6	2.5E-7	8.9E-6
	GOTERM_MF_FAT	transcription factor binding	65	34.8	8.9E-7	5.6E-5
	GOTERM_MF_FAT	protein dimerization activity	35	18.7	1.2E-6	6.1E-5
	GOTERM_MF_FAT	transcription factor activity	111	59.4	7.8E-5	1.8E-3
Non_Sumoylation						
	PFAM	KRAB	247	14.3	4.5E-9	6.8E-6
	PFAM	KRAB box	224	12.9	5.1E-8	3.9E-5
O_GlcNAc						
	SP_COMMENT_TYPE	PTM	26	65.0	1.1E-7	2.0E-6
	SP_PIR_KEYWORDS	phosphoprotein	37	92.5	9.9E-9	8.0E-7
	SP_PIR_KEYWORDS	acetylation	17	42.5	4.7E-4	1.3E-2

Table S3 - Associations between property-based subgroups

Property 1	Property 2	Pvalue	Benjamini	Corr.
Phosphorylation	Acetylation	1.546e-10	4.328e-09	0.19
Phosphorylation	Methylation	1.614e-10	2.260e-09	0.13
Phosphorylation	Ubiquitination	2.679e-10	2.501e-09	0.19
Methylation	Ubiquitination	2.788e-10	1.951e-09	0.20
Methylation	Acetylation	2.812e-10	1.575e-09	0.20
DNA_Binding	Methylation	3.125e-10	1.458e-09	-0.16
DNA_Binding	Ubiquitination	3.3975e-10	1.359e-09	-0.28
Acetylation	Ubiquitination	3.595e-10	1.258e-09	0.29
Acetylation	Sumoylation	4.009e-09	1.247e-08	0.13
Ubiquitination	Sumoylation	6.329e-08	1.772e-07	0.12
DNA_Binding	Acetylation	8.483e-08	2.159e-07	-0.12
Phosphorylation	Sumoylation	9.969e-06	2.326e-05	0.08
Methylation	O_GlcNAc	1.176e-05	2.534e-05	0.11
Acetylation	O_GlcNAc	0.000	0.000	0.08
Ubiquitination	O_GlcNAc	0.004	0.007	0.07
PPI	Sumoylation	0.012	0.021	0.07
Methylation	Sumoylation	0.015	0.025	0.05
Phosphorylation	O_GlcNAc	0.028	0.044	0.05
PPI	Ubiquitination	0.090	0.131	0.05
DNA_Binding	Phosphorylation	0.093	0.131	-0.04
Sumoylation	O_GlcNAc	0.279	0.371	0.02
DNA_Binding	PPI	0.343	0.436	-0.03
PPI	Phosphorylation	0.370	0.450	0.03
PPI	Acetylation	0.551	0.644	-0.02
DNA_Binding	O_GlcNAc	0.630	0.705	-0.01
DNA_Binding	Sumoylation	0.753	0.819	0.09
PPI	O_GlcNAc	0.816	0.846	0.07
PPI	Methylation	0.876	0.876	-0.07

Table S4 - Output from enrichment analysis of data from Tuomela *et al.*

(Here you have to set cutoff on Benjamini to 0.2 to get the least significant results.)

Please enter the path of the reference database (xls, with title row): Main_table.xls
Please enter the path of the test set (xls, with title row): Tuomela_data.xls
Please enter the column number for TFs in your table (starting at 0): 2

Category	Term	Observed	Expected	Pvalue	Benjamini	MCC
Cluster1						
	Sumoylation	2	0	4.84E-02	1.93E-01	0.062
Cluster2						
	There is no overrepresented item for this Gene/Protein list					
Cluster3						
	There is no overrepresented item for this Gene/Protein list					
Cluster4						
	There is no overrepresented item for this Gene/Protein list					
Cluster5						
	There is no overrepresented item for this Gene/Protein list					
Cluster6						
	O_GlcNAc	3	0	9.69E-03	7.75E-02	0.086
	Ubiquitination	16	9	1.58E-02	6.31E-02	0.058
	Methylation	9	4	2.35E-02	6.27E-02	0.059
Cluster7						
	There is no overrepresented item for this Gene/Protein list					
Cluster8						
	Ubiquitination	17	9	1.36E-03	1.09E-02	0.074
	PPI	10	5	2.40E-02	9.59E-02	0.070
	O_GlcNAc	2	0	6.87E-02	1.83E-01	0.054

	PF00623(RNA_pol_Rpb1_2)	2	0	3.20E-04	1.17E-01	0.249
	PF05000(RNA_pol_Rpb1_4)	2	0	3.20E-04	7.77E-02	0.249
	PF01344(Kelch_1)	2	0	3.20E-04	5.83E-02	0.249
	PF04997(RNA_pol_Rpb1_1)	2	0	3.20E-04	4.66E-02	0.249
	PF04998(RNA_pol_Rpb1_5)	2	0	3.20E-04	3.88E-02	0.249
Cluster9						
	There is no overrepresented item for this Gene/Protein list					
Cluster10						
	There is no overrepresented item for this Gene/Protein list					
Cluster1,2,3						
	PPI	9	4	1.58E-02	1.26E-01	0.072
Cluster4,5,6						
	Ubiquitination	43	29	1.48E-03	1.18E-02	0.074
	Methylation	21	12	1.03E-02	4.11E-02	0.061
	Sumoylation	12	6	2.98E-02	7.93E-02	0.054
	O_GlcNAc	4	1	4.53E-02	9.07E-02	0.052
	Acetylation	36	29	1.04E-01	1.66E-01	0.037

	PF00554(RHD)	4	0	2.04E-04	1.49E-01	0.146
Cluster7,8,9,10						
	DNA_Binding	28	38	7.49E-03	5.99E-02	-0.062
	Ubiquitination	38	28	1.32E-02	5.29E-02	0.058

Table S5 - Output from enrichment analysis of data from Lawrence *et al.*

```
Please enter the path of the reference database (xls, with title row): Main_table.xls
Please enter the path of the test set (xls, with title row): Lawrence_data.xls
Please enter the column number for TFs in your table (starting at 0): 0
```

Category	Term	Observed	Expected	Pvalue	Benjamini	MCC
I						
	Methylation	4	0	5.47E-03	4.38E-02	0.078
II						
	Acetylation	21	12	1.67E-03	1.33E-02	0.073
	Ubiquitination	20	12	6.61E-03	2.64E-02	0.063
	Methylation	11	5	1.23E-02	3.28E-02	0.062
	O_GlcNAc	3	0	1.89E-02	3.78E-02	0.073
IIIAB						
	Acetylation	27	14	5.47E-06	4.38E-05	0.102
	Ubiquitination	27	14	6.39E-06	2.56E-05	0.101
	Methylation	15	6	2.79E-04	7.44E-04	0.091
	Sumoylation	9	3	2.28E-03	4.56E-03	0.081
	DNA_Binding	12	19	5.45E-03	8.71E-03	-0.065

	PF08214(KAT11)	2	0	2.54E-04	4.62E-02	0.248
	PF02135(zf-TAZ)	2	0	2.54E-04	3.69E-02	0.248
	PF00514(Arm)	2	0	2.54E-04	3.08E-02	0.248
	PF02172(KIX)	2	0	2.54E-04	2.64E-02	0.248
	PF00439(Bromodomain)	4	0	4.78E-04	4.35E-02	0.132
IIIB						
	Sumoylation	5	1	3.51E-03	2.80E-02	0.085
I+IIIAB						
	Ubiquitination	32	16	2.69E-07	2.15E-06	0.114
	Acetylation	30	16	6.44E-06	2.58E-05	0.101
	Methylation	19	7	7.94E-06	2.12E-05	0.114
	Sumoylation	10	3	1.82E-03	3.64E-03	0.082
	DNA_Binding	15	22	9.56E-03	1.53E-02	-0.061

	PF00856(SET)	5	0	1.67E-05	1.21E-02	0.178
	PF05964(FYRN)	3	0	2.38E-05	8.67E-03	0.243
	PF05965(FYRC)	3	0	2.38E-05	5.78E-03	0.243
	PF00628(PHD)	7	1	4.70E-05	8.56E-03	0.136
	PF13771(zf-HC5HC2H)	3	0	3.17E-04	4.61E-02	0.168
	PF06001(DUF902)	2	0	3.41E-04	4.13E-02	0.230
	PF09030(Creb_binding)	2	0	3.41E-04	3.54E-02	0.230
	PF08214(KAT11)	2	0	3.41E-04	3.10E-02	0.230
	PF02135(zf-TAZ)	2	0	3.41E-04	2.76E-02	0.230
	PF00514(Arm)	2	0	3.41E-04	2.48E-02	0.230
	PF02172(KIX)	2	0	3.41E-04	2.25E-02	0.230
II+IIIAB						
	Acetylation	48	26	1.83E-08	1.46E-07	0.126
	Ubiquitination	47	27	2.08E-07	8.31E-07	0.117
	Methylation	26	11	1.21E-05	3.23E-05	0.110
	Sumoylation	14	5	1.14E-03	2.28E-03	0.082
	O_GlcNAc	5	1	7.03E-03	1.12E-02	0.078

	PF00856(SET)	6	0	1.21E-05	8.80E-03	0.164
	PF13771(zf-HC5HC2H)	4	0	4.90E-05	1.78E-02	0.175
	PF05964(FYRN)	3	0	1.04E-04	2.52E-02	0.189
	PF05965(FYRC)	3	0	1.04E-04	1.89E-02	0.189
	PF00628(PHD)	8	1	1.78E-04	2.58E-02	0.114

Table S6 - Output from enrichment analysis of data from Vaquerizas *et al.*

```
Please enter the path of the reference database (xls, with title row): Main_table.xls
Please enter the path of the test set (xls, with title row): Vaquerizas_data.xls
Please enter the column number for TFs in your table (starting at 0): 0
```

Category	Term	Observed	Expected	Pvalue	Benjamini	MCC
General						
	DNA_Binding	126	85	1.36E-10	1.09E-09	0.166

	PF13465(zf-H2C2_2)	54	31	6.90E-06	5.02E-03	0.108
Specific						
	DNA_Binding	306	205	1.92E-10	1.53E-09	0.280
	Sumoylation	57	31	1.85E-06	7.39E-06	0.115

	PF00104(Hormone_recep)	28	7	2.32E-11	1.69E-08	0.179
	PF00105(zf-C4)	27	7	4.29E-11	1.56E-08	0.176
	PF00170(bZIP_1)	18	5	3.56E-07	8.64E-05	0.135
	PF01352(KRAB)	16	40	9.20E-07	1.67E-04	-0.103
	PF00010(HLH)	30	13	6.26E-06	9.11E-04	0.112
	PF00178(Ets)	13	4	1.41E-04	1.71E-02	0.099
Unknown						
	DNA_Binding	702	486	2.82E-10	2.26E-09	0.459
	Ubiquitination	229	355	3.19E-10	1.28E-09	-0.263
	Methylation	105	149	1.68E-07	4.47E-07	-0.116
	Sumoylation	52	75	2.34E-04	4.69E-04	-0.082
	PPI	146	172	1.48E-03	2.37E-03	-0.091
	Acetylation	317	350	1.95E-03	2.60E-03	-0.070
	Phosphorylation	698	713	1.68E-02	1.92E-02	-0.054

	PF00046(Homeobox)	121	72	1.82E-10	1.33E-07	0.174
	PF01352(KRAB)	200	96	2.11E-10	7.66E-08	0.324
	PF00096(zf-C2H2)	140	83	2.14E-10	5.19E-08	0.190
	PF12796(Ank_2)	0	17	2.93E-10	5.34E-08	-0.122
	PF13465(zf-H2C2_2)	318	177	3.19E-10	4.65E-08	0.346
	PF00076(RRM_1)	1	15	1.14E-07	1.38E-05	-0.108
	PF13912(zf-C2H2_6)	45	25	2.82E-07	2.94E-05	0.118
	PF02023(SCAN)	32	19	1.50E-04	1.36E-02	0.087
	PF00538(Linker_histone)	9	3	2.38E-04	1.92E-02	0.083
